# Evolution of T-cell clonality in a patient with Ph-negative acute lymphocytic leukemia occurring after interferon and imatinib therapy for Ph-positive chronic myeloid leukemia

**DOI:** 10.1186/1756-8722-3-14

**Published:** 2010-04-09

**Authors:** Liang Wang, Kanger Zhu, Xianfeng Zha, Shaohua Chen, Lijian Yang, Si Chen, Yangqiu Li

**Affiliations:** 1 Department of Hematology, First Affiliated Hospital, Jinan University, Guangzhou, 510632, PR China; 2 Institute of Hematology, Medical College, Jinan University, Guangzhou, 510632, PR China; 3 Key Laboratory for Regenerative Medicine of Ministry of Education, Jinan University, Guangzhou, 510632, PR China

## Abstract

**Introduction:**

The development of Philadelphia chromosome (Ph) negative acute leukemia/myelodysplastic syndrome (MDS) in patients with Ph-positive chronic myeloid leukemia (CML) is very rare. The features of restrictive usage and absence of partial T cell clones have been found in patients with CML. However, the T-cell clonal evolution of Ph-negative malignancies during treatment for CML is still unknown.

**Objective:**

To investigate the dynamic change of clonal proliferation of T cell receptor (TCR) Vα and Vβ subfamilies in one CML patient who developed Ph-negative acute lymphoblastic leukemia (ALL) after interferon and imatinib therapy.

**Methods:**

The peripheral blood mononuclear cells (PBMC) samples were collected at the 3 time points (diagnosis of Ph-positive chronic phase (CP) CML, developing Ph-negative ALL and post inductive chemotherapy (CT) for Ph-negative ALL, respectively). The CDR3 size of TCR Vα and Vβ repertoire were detected by RT-PCR. The PCR products were further analyzed by genescan to identify T cell clonality.

**Results:**

The CML patient who achieved complete cytogenetic remission (CCR) after 5 years of IFN-α therapy suddenly developed Ph-negative ALL 6 months following switch to imatinib therapy. The expression pattern and clonality of TCR Vα/Vβ T cells changed in different disease stages. The restrictive expression of Vα/Vβ subfamilies could be found in all three stages, and partial subfamily of T cells showed clonal proliferation. Additionally, there have been obvious differences in Vα/Vβ subfamily of T cells between the stages of Ph-positive CML-CP and Ph-negative ALL. The Vα10 and Vβ3 T cells evolved from oligoclonality to polyclonality, the Vβ13 T cells changed from bioclonality to polyclonality, when Ph-negative ALL developed.

**Conclusions:**

Restrictive usage and clonal proliferation of different Vα/Vβ subfamily T cells between the stages of Ph-positive CP and Ph-negative ALL were detected in one patient. These changes may play a role in Ph- negative leukemogenesis.

## Introduction

Chronic myeloid leukemia (CML) is genetically characterized by the presence of the reciprocal translocation t (9; 22) (q34; q11), resulting in a BCR/ABL gene fusion on the derivative chromosome 22 called the Philadelphia chromosome (Ph). Blastic transformation (BT) remains a dire outcome of CML patients with a poor prognosis. Non-random additional chromosome abnormalities accompanied by Philadelphia chromosome can be detected in 60-80% of cases in BT[1]. Recently, however, the development of chromosomal abnormalities in Ph-negative cells[2] and isolated instances of Ph-negative acute leukemia or high-risk MDS during treatment for CML have been reported [2-10]. The clonal origin of Ph-negative leukemic clone is still unknown,. It is possible that it may originate from a de novo leukemic stem cell (malignant clone) due to therapy related toxicity such as interferon, imatinib or other agents.

T cell immunodeficiency was suggested to play an important role in tumor patients by facilitating the expansion of a malignant clone[11,12]. Clonally expanded T-cells were identified in peripheral blood or tumor infiltrating T-cells (TIL), which are thought to play a pivotal role in the adaptive immune responses by recognizing antigen- derived peptides bound to MHC molecules. The clonality of T-cells could be identified by analysis of CDR3 size of 24 TCR Vβ genes using RT-PCR and genescan, which is called "immunoscope"[13,14]. Several studies on TCR Vβ repertoire showed that skewed expression of TCR Vβ subfamilies is a common feature in leukemia patients [15-18]. Clonally expanded T cells with restricted TCR Vβ usage can recognize tumor cells in patients with both solid tumors and leukemia [16,19,20].

It has been reported that leukemia-associated antigen can induce specific clonal expansion of host T-cells or the allogeneic T-cells. These activated T-cells have been shown to display potential cytotoxic activity against primary leukemic cells. Thus, it may be useful for eradication of minimal residual leukemic cells by activating autologous or allogeneic cytotoxic cells. In particular, specific CTLs may be a promising tool in the treatment of myelogenous leukemia [16,17,21]. Our previous study showed that clonal expansion of T-cells could be induced by CML associated antigen[16]. However, it is unclear how the clonally expanded TCR Vβ T-cells in CML patients are related to the development of Ph-negative acute leukemia. In the present study, we have used reverse transcription polymerase chain reaction (RT-PCR) and the genescan analysis to assay for TCR Vα and Vβ gene utilization and clonal expansion in a patient who developed Ph-negative acute lymphoblastic leukemia while in CML complete remission following interferon and imatinib mesylate therapy.

## Methods

### Case history

A 10-year-old female presented to our hospital in October 2000 because of excessive tiredness, epistaxis and weight loss. Examination revealed moderate hepatosplenomegaly, and a blood count showed hemoglobin 102 g/L, white cell count 179 × 10^9^/L, blasts 1%, promyelocytes 8%, myelocytes 10%, metamyelocytes 29%, eosinophils 1%, basophils 7%, bands 16%, polymorphs 26%, lymphocytes 2% and platelets 917 × 10^9^/L. Leukocyte alkaline phosphatase was 11. Bone marrow examination was consistent with chronic phase CML (CML-CP). Cytogenetic studies showed 25/25 cells with 46, XX, t(9;22), t(11;18), der(16), t(16;?) by R-banding technique. Fluorescence in situ hybridization (FISH) and reverse transcription polymerase chain reaction (RT-PCR) studies for BCR/ABL fusion gene were positive. She received interferon-alpha (IFN-α) combined with hydroxyurea therapy. Hydroxyurea was discontinued three weeks later, when white cell count decreased to 5.7 × 10^9^/L, and spleen and liver became non-palpable. Treatment with IFN-α was commenced at a dose of 1.5 million-units (MU)/day. BCR/ABL fusion gene remianed positive (90%~100%) by FISH analysis, which was performed once or twice per year from 2001 to 2005. In May 2005, we boosted the dose of IFN-α from 1.5 to 3 MU/day. Unfortunately, the patient failed to tolerate full-dose IFN-α due to leukopenia (1 × 10^9^/L) complicated with fever. We discontinued IFN-α therapy for 3 months. After this, the dose of IFN-α ranged from 1.5 to 3 MU per week according to white cell count. In January 2006, FISH analysis revealed that the patient achieved complete cytogenetic remission (CCR). At this time, IFN-α was stopped, and imatinib mesylate (IM, 400 mg/d) was given instead according to the patient's selection. BCR/ABL fusion gene was detected using FISH analysis of marrow samples in March, May and August 2006.

In October 2006, the patient was admitted to our department again due to sudden onset of overall osteodynia, especially both in lower extremities, sternum and ribs. A blood count showed hemoglobin 102 g/L, white cell count 5.72 × 10^9^/L, myelocytes 1%, bands 14%, polymorphs 24%, monocytes 12%, eosinophils 1%, lymphocytes 48% and platelets 75 × 10^9^/L. Bone marrow smear revealed 95% blasts that expressed CD34, HLA-DR and the lymphoid antigens CD19, CD20 and CD10. The blasts were myeloperoxidase negative by cytochemistry staining, and cytogenetic analysis showed 25/25 cells with 46, XX. Repeat FISH analysis of this sample confirmed 200/200 metaphase cells to be Ph-negative. After receiving two courses of induction chemotherapy consisting of CMOP regimen (cyclophosphamide, mitoxantone, vincristine and prednisone) and FLAG regimen (fludarabine, cytoarabine and granulocyte-cloning stimulating factor), respectively, the patient achieved complete remission (CR). Unfortunately bone marrow aspirate performed four weeks later showed relapse with 67% lymphoblasts. The karyotype was still normal, and BCR/ABL fusion gene was still negative by FISH. The patient was treated palliatively and died of pulmonary invasive fungi infection in June 2007.

### Samples

After the patient's consent, the bone marrow and peripheral blood samples were collected in three different disease stages of Ph-positive CP-CML, Ph-negative ALL and post two courses of chemotherapy (CT) for Ph-negative ALL, respectively.

### Cytogenetic, FISH and RT-PCR analysis for BCR/ABL detection

Karyotype analyses were performed by R-banding technique. FISH was performed using LSI·bcr/abl dual color probe (Vysis) that identified BCR/ABL rearrangement derived from t (9; 22) (q34; q11.2). Three primers of RT-PCR analyses for BCR/ABL detection were listed in Table 1, and PCR was performed as described by Kawasaki ES et al [22].

**Table 1:** The sequence of primers used for detection of BCR/ABL rearrangement

Primers	Sequences
CML 1 (upstream)	5'-GGAGCTGCAGATGCTGACCAAC-3'
CML 2 (downstream)	5'-TCAGACCCTGAGGCTCAAAGTC-3'
CML 3 (upstream)	5'-CGCATGTTCCGGGACAAAAGC-3'

### Peripheral blood mononuclear cells (PBMC) isolation, RNA isolation and cDNA synthesis

PBMC were isolated by Ficoll-Hypaque gradient centrifugation. RNA was extracted from the PBMC samples according to the manufacturer's recommendations (Trizol, Gibco, USA): The quality of RNA was analyzed in 0.8% agarose gel stained with ethidium bromide. Two μg RNA was reversely transcribed into the first single-strand cDNA with random hexamer primers, using reverse transcriptase, Superscript II Kit (Gibco, USA). The quality of cDNA was confirmed by RT-PCR for β2 microglubin gene amplification.

### RT-PCR for TCR Vα and TCR Vβ subfamily amplification

29 sense TCR Vα primers and a single TCR Cα reverse primer, or 24 TCR Vβ sense primers and a single TCR Cβ primer were used in unlabeled PCR for amplification of the TCR Vα and Vβ subfamilies respectively[23]. Subsequently, a runoff PCR was performed with fluorescent primers labelled at 5' end with the FAM fluorophore (Cα-FAM or Cβ-FAM) purchased from TIB MOLBIOL GmbH, Berlin, Germany. PCR was performed as described by Puisieux I et al and our previous studies[16,23,24]. Aliquots of the cDNA (1 μl) were amplified in 25 μl reactions with one of the 29 Vα primers and a Cα primer or one of 24 Vβ primers and a Cβ primer. The final reaction mixture contained 0.5 μM sense primer and antisense primer, 0.1 mM dNTP, 1.5 mM MgCl_2_, 1×PCR buffer and 1.25 U Taq polymerase (Promega, USA). The amplification was performed on a DNA thermal cycler (BioMetra, Germany). After 3 min denaturation at 94°C, 40 PCR cycles were performed, each cycle consisting of 94°C for 1 min, 60°C for 1 min and 72°C for 1 min, and a final 7 min elongation at 72°C. Then, the products were stored at 4°C.

### Genescan analysis for TCR Vα and TCR Vβ subfamily clonality analysis

Aliquots of the unlabeled PCR products (2 μl) were subjected to a cycle of runoff reaction with fluorophore-labelled Cα-fam or Cβ-fam primer respectively. The labelled runoff PCR products (2 μl) were heat-denatured at 94°C for 4 min with 9.5 μl formamide (Hi-Di Formamide, ABI, USA) and 0.5 μl of Size Standards (GENESCAN™-500-LIZ™, Perkin Elmer, ABI), the samples were then loaded on 3100 POP-4™ gel (Performance Optimized Polymer-4, ABI, USA) and resolved by electrophoresis in 3100 DNA sequencer (ABI, Perkin Elmer) for size and fluorescence intensity determination using Genescan software[16,23,24].

## Results

### Genetic feature of the CML case

**Figure 1:**
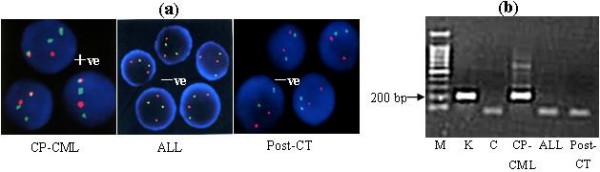
**The results of FISH and RT-PCR analyses of marrow samples aspirated during different disease stage in a patient with CML**. CP-CML, the sample from chronic phase (October 2000); ALL, from the disease stage of acute lymphoblastic leukemia (October 2006); Post-CT, from post-induction chemotherapy for ALL (December 2006). + ve, BCR/ABL positive; - ve, BCR/ABL negative. (a) FISH analysis; (b) RT-PCR analysis. Lane M, 100 bp molecular weight ladder; Lane K, K562 200 bp b3/a2 BCR/ABL positive control; Lane C: negative control; Lane CP-CML, 200 bp b3/a2 products; Lane ALL and Post-CT, BCR/ABL negative.

Clinical, cytogenetic and molecular features of different disease stage in this patient were listed in Table 2. Cytogenetic studies showed 25/25 cells with 46, XX, t(9;22), t(11;18), der(16), t(16;?) by R-banding technique at the diagnosis of CP-CML in October 2000. FISH and RT-PCR studies for BCR/ABL fusion gene were also positive. In October 2006, when the patient was diagnosed to have ALL, she had normal karyotype and negative FISH and RT-PCR studies for BCR/ABL (Figure 1).

**Table 2:** Clinical, cytogenetic and molecular features of a patient with Ph-positive CML who developed Ph-negative acute lymphoblastic leukemia after IFN-α and imatinib mesylate therapy

Date	Disease stage (Treatment)	Karyotype	BCR/ABL	
			
			FISH	RT-PCR
8/10/2000	CP	46, XX, t(9;22), t(11;18), der(16), t(16;?)[25]	+ve(95%)	+ve
3/3/2001	CP(IFN-α)	ND	+ve(95%)	ND
21/12/2001	CP(IFN-α)	45, XX,-22,16q+ t(11;18)[1]/45, XX,-22,16q+ t(9;22)t(11;18)[1]/46, XX,-22,16q+ t(9;22)t(11;18)[18]/46, XX [5]	+ve(90%)	ND
28/11/2002	CP(IFN-α)	ND	+ve(100%)	ND
28/11/2003	CP(IFN-α)	ND	+ve(90%)	ND
6/5/2004	CP(IFN-α)	ND	+ve(90%)	ND
1/6/2005	CP(IFN-α)	ND	+ve(75%)	ND
27/1/2006	CCR(IFN-α)	ND	-ve	ND
8/3/2006	CCR (IM)	ND	-ve	ND
11/5/2006	CCR (IM)	ND	-ve	ND
9/8/2006	CCR (IM)	ND	-ve	ND
8/10/2006	Ph-negative ALL	46, XX [25]	-ve	-ve
18/12/2006	Post CT for ALL	46, XX [25]	-ve	-ve

CP: chronic phase; ALL: acute lymphoblastic leukemia; CCR: complete cytogenetic response; Hu: hydroxyurea; IM: imatinib mesylate; CT: chemotherapy; +ve: positive; -ve: negative; ND: not done.

### TCR Vα and TCR Vβ repertoire in PB T-cells

**Figure 2:**
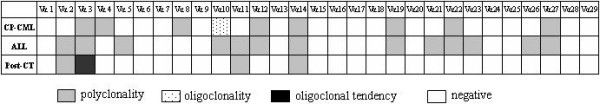
**Distributions and clonality of TCR Vα subfamilies in a CML patient with different disease stages (CP-CML, ALL and Post-CT)**.

**Figure 3:**
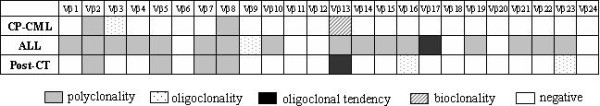
**Distributions and clonality of TCR Vβ subfamilies in a CML patient with different disease stages (CP-CML, ALL and Post-CT)**.

In different disease stage, the expression pattern of Vα and Vβ repertoires was different. Only 9, 13 and 4 TCR Vα subfamilies were detected in PBMCs from the disease stage of CP-CML, ALL and post CT for ALL, respectively. TCR Vβ subfamilies 4, 18 and 7 were detected in PBMCs from the disease stage of CP-CML, ALL and post CT, respectively, whereas almost all Vα and Vβ subfamilies could be detected in healthy controls. When patient developed ALL, 6 TCR Vα and 14 TCR Vβ subfamilies were newly expressed, and 2 TCR Vα (Vα4 and Vα8) subfamilies disappeared (Figure 2 and 3).

### The clonality of TCR Vα/Vβ subfamily T-cells in different disease stages

**Figure 4:**
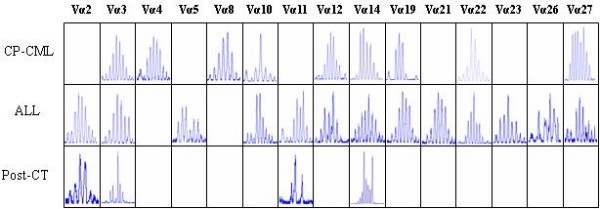
**The results of genescan of TCR Vα subfamilies in a CML patient with different disease stages (CP-CML, ALL and Post-CT)**.

**Figure 5:**
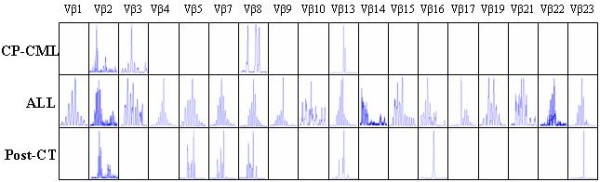
**The results of genescan of TCR Vβ subfamilies in a CML patient with different disease stages (CP-CML, ALL and Post-CT)**.

Polyclonality of T cells representing random rearrangement of TCR genes were detected in most TCR Vα/Vβ subfamily in PBMCs of the patient in different disease stages. Clonal expansion of TCR Vβ repertoire could be found in some TCR Vβ subfamilies, which displayed different pattern between CT and ALL. Vβ13 or Vβ9 and Vβ17 were identified at the stage of CT and ALL respectively. More oligoclonal TCR Vβ T cells were detected after CT for ALL in the patient (Figure 4 and 5).

## Discussion

Isolated instances of Ph-negative acute leukemia or high-risk MDS have been observed in the course of interferon-α [4,9] and imatinib therapy[2,3] or post hematopoietic stem cell transplantation[5] for Ph-positive CML. In the present study, we reported a similar case which developed Ph-negative acute lymphoblastic leukemia following imatinib therapy for 6 months. It was thought that the Ph-negative leukemic cells might originate from a new malignant clone rather than previous Ph-positive clone [25]. The cause of this phenomenon remains unclear. In the present study, we characterized the T-cell repertoires between the stages of CML-CP and Ph- negative ALL. Our previous studies showed that the clonally expanded T cells were associated with a leukemia associated antigen [16]. The newly generated malignant clone might express different leukemia specific or associated antigen, which may induce different response of TCR repertoire pattern. It would be interesting to detect the evolution of T-cell clonality in the patient at different disease status.

The features of restrictive usage and absence of partial T cell clones could be found in patients with CML [26], which indicate deficiency of cellular immunity in CML patients. However, on the other hand, anti-CML cytotoxic T-cell clones were also identified in patients with CML. These specific CTLs could be detected in T cells from peripheral blood of CML patients or autologous T cells inducted by bcr-abl peptide and so on [15]. In the present study, the TCR Vα and Vβ distribution and T cell clonality were analyzed by RT-PCR-genescan technique in a CML patient with different disease stages. As expected, there are marked difference in the expressional number of TCR Vα/Vβ between the disease stage of CP and that of Ph-negative ALL. Only 9 of all 29 Vα and 4 of all 24 Vβ subfamilies could be detected at the time of CP, while 13/29 Vα and 18/24 Vβ subfamilies could be found at the time of Ph-negative ALL. Additionally, decreased number of TCR Vα/Vβ subfamilies was detected post chemotherapy. The distinct distribution of clonal T cells were also detected in different disease stage, the pattern changes in the clonally expanded T cells between the chronic phase CML and Ph-negative acute lymphoblastic leukemia might represent the change of the host cellular immune response. Obviously, the predominant usage of TCR Vβ subfamilies were TCR Vβ3 and Vβ13 in oligoclonal expanded T cells from CML-CP, while the usage pattern changed to TCR Vβ9 and Vβ17, when acute lymphoblastic leukemia developed. This phenomenon may be caused by leukemic antigen variation due to leukemia clonal change from a Ph-positive clone to a Ph-negative clone. Although the antigenic peptides leading to clonal T-cell selection in CML are unknown, the change of TCR Vβ clones might provide the information for host immune response. After two courses of chemotherapy for Ph-negative ALL, the decrease of TCR subfamilies including Vα and Vβ were possibly induced by chemotherapy. There are two possible mechanisms to interpret the occurrence of oligoclonal T cells, including immunity response to novel leukemic antigen and immunosuppression by chemotherapy.

In the present study, oligoclonally- expanded T cells seem unmarked, when the TCR Vα repertoire analysis was used. Oligoclonally- expanded T cells was found only in Vα10 subfamily in CML-CP, which changed to polyclonally expanded T cells in ALL. It may indicate that the polyclonally expanded pattern was a common feature in TCR Vα subfamily T cells. Thus, the TCR Vβ repertoire analysis was thought more sensitive for detecting clonally expanded T cells in immune response, at least, in the present CML case.

To our knowledge, this is the first investigation of T-cell clonal changes in Ph-negative ALL and Ph-positive CML in the same patient. More cases of secondary Ph-negative leukemia/MDS are needed to better characterize the clonal expansion and evolution of T-cell repertoire.

## Conclusions

Restrictive usage and clonal proliferation of different Vα/Vβ subfamily T cells between the stages of Ph-positive CP and Ph-negative ALL were detected in one patient. These changes may play a role in Ph- negative leukemogenesis.

## Competing interests

The authors declare that they have no competing interests.

## Authors' contributions

LW, YQL and KEZ were responsible for study design and data management. LW and XFZ collected samples, recorded all clinical data, and detected the CDR3 size of TCR Vα and Vβ repertoire RT-PCR. LW, SHC and SC carried out genescan. YQL, KEZ and LJY participated together with LW in editing the manuscript. All authors read and approved the final manuscript.
